# Incidence and cost of haemolytic uraemic syndrome in urban China: a national population-based analysis

**DOI:** 10.1186/s12882-022-02746-2

**Published:** 2022-03-30

**Authors:** Jingnan Feng, Ke Xu, Xinmiao Shi, Lu Xu, Lili Liu, Fang Wang, Xuhui Zhong, Guozhen Liu, Jinxi Wang, Pei Gao, Jie Ding, Shengfeng Wang, Siyan Zhan

**Affiliations:** 1 Department of Epidemiology and Biostatistics, School of Public Health, Peking University Health Science Center, Haidian District, No.38, Xueyuan Rd, Beijing, China; 2 Department of Pediatric, Peking University First Hospital, No.1 Xi An Men Da Jie, Beijing, China; 3 Peking University Health Information Technology Co. Ltd, Beijing, China; 4 Shanghai Songsheng Business Consulting Co. LTD, Beijing, China; 5 Research Center of Clinical Epidemiology, Peking University Third Hospital, Beijing, China

**Keywords:** Haemolytic uraemic syndrome, Incidence, Cost, Insurance database

## Abstract

**Background:**

Haemolytic uraemic syndrome (HUS) is a severe syndrome that causes a substantial burden for patients and their families and is the leading cause of acute kidney injury in children. However, data on the epidemiology and disease burden of HUS in Asia, including China, are limited. We aimed to estimate the incidence and cost of HUS in China.

**Methods:**

Data about HUS from 2012 to 2016 were extracted from the Urban Employee Basic Medical Insurance (UEBMI) and Urban Resident Basic Medical Insurance (URBMI) databases. All cases were identified by ICD code and Chinese diagnostic terms. The 2016 national incidence rates were estimated and stratified by sex, age and season. The associated medical costs were also calculated.

**Results:**

The crude incidence of HUS was 0.66 per 100,000 person-years (95% CI: 0.35 to 1.06), and the standardized incidence was 0.57 (0.19 to 1.18). The incidence of HUS in males was slightly higher than that in females. The age group with the highest incidence of HUS was patients < 1 year old (5.08, 95% CI: 0.23 to 24.87), and the season with the highest incidence was autumn, followed by winter. The average cost of HUS was 2.15 thousand US dollars per patient, which was higher than the national average cost for all inpatients in the same period.

**Conclusions:**

This is the first population-based study on the incidence of HUS in urban China. The age and seasonal distributions of HUS in urban China are different from those in most developed countries, suggesting a difference in aetiology.

**Supplementary Information:**

The online version contains supplementary material available at 10.1186/s12882-022-02746-2.

## Introduction

Haemolytic uraemic syndrome (HUS), which was first described in the 1950s, is a severe disease. All patients with HUS present with microangiopathic haemolytic anaemia, thrombocytopenia, and acute kidney injury. The presence of lesions that are consistently restricted to the kidneys is key to distinguishing HUS from thrombotic thrombocytopenic purpura (TTP), which is another form of thrombotic microangiopathy (TMA) in which brain lesions prevail and are caused by a severe deficiency (< 10%) in the expression of ADAMTS13 (A Disintegrin And Metalloproteinase with a ThromboSpondin type 1 motif, member 13) [1]. Most HUS cases are caused by Shiga toxin-producing Escherichia coli (STEC) infections. STEC accounts for 70 to 90% of all incident cases of paediatric HUS [2]. The remaining HUS cases are originally diagnosed as “atypical HUS (aHUS)”. Currently, aHUS is classified as *Streptococcus pneumoniae*-HUS, influenza-HUS, alternative complement pathway dysregulation-HUS, complement-independent HUS (*Cobalamin C*, *DGKE* or *INF2* mutation), HUS with coexisting disease (transplantation, autoimmune disorders, drugs, malignant hypertension, malignancy/cancer chemotherapy), and idiopathic HUS (unclear aetiology) [3, 4]. HUS is the leading cause of acute kidney injury in children. Although the HUS-related mortality rate in children in industrialized countries has decreased, 3 to 5% of patients still die during the acute phase of STEC-HUS, and approximately 12% of these patients progress to end-stage renal disease (ESRD). Most patients with aHUS have a poor outcome; up to 50% ~ 60% of these patients progress to ESRD or develop irreversible brain damage, and 25% die during the acute phase of the disease [5]. In addition, patients with HUS incur high medical expenses, and the total direct cost was reported to be 17,553.39 US dollars per patient in 2005 in Argentina [6]. Since 2011, the treatment of HUS has been revolutionised by the introduction of the anti-C5 monoclonal antibody eculizumab. More than 80% of aHUS patients achieved a TMA event-free status after receiving eculizumab, which is considered a first-line treatment for complement-mediated HUS in many other countries but is still not available in China [7]. 

The annual incidence of STEC-HUS varies by year and region. Prior to 2000, the overall incidence of STEC-HUS was estimated to be 2.1 cases per 100,000 persons/yr, with a peak incidence in children who were younger than 5 yr (6.1 per 100,000/yr) and the lowest incidence in adults who were between 50 and 59 yr of age (0.5 per 100,000/yr) [8]. The overall annual rate of notified HUS in Australia between 2000 and 2010 was 0.07 cases per 100,000 per year [9]. Among patients of all ages, the annual incidence of aHUS ranges from 0.23 to 1.9 per million people [10].

Only a few single-centre case series about children with HUS have been conducted in China, and relevant data across Asia are also limited. HUS was included in the Target Rare Diseases List (TRDL) in 2017 and was one of the top 10 rare diseases with the highest rates of readmission in China [11]. Because the epidemiology of HUS in China is unknown, we conducted a retrospective analysis of the epidemiological characteristics and the associated medical costs based on medical insurance database records of patients with HUS in China.

## Materials and methods

### Data source

There are two main health insurance programs in the urban areas of China: The Urban Employee Basic Medical Insurance (UEBMI) for urban working and retired employees and the Urban Residence Basic Medical Insurance (URBMI) for urban residents without formal employment. The data regarding HUS were obtained from the claims information in these two databases. Briefly, the insured individuals’ sociodemographic characteristics (age, sex), the International Classification of Diseases (ICD) code, the names of the major and secondary disease, and the total medical expenses were extracted for analysis. The study protocol was approved by the ethics review committee of the Peking University Health Science Center, and the need to obtain informed consent was waived (IRB00001052-18012).

### Study population

A retrospective national population-based study in 16 provinces of mainland China was performed from January 1^st^, 2012, to December 31^th^, 2016 (Supplementary table 1). Provinces were excluded from this study due to the following reasons: missing information on ICD codes or diagnostic terms; reporting policy exemptions; only one insurance type available; missing information or abnormal data reporting for crucial information, e.g., the primary diagnosis; short history of electronic records (< five years). The claims data used in this study were all anonymous.

### HUS case identification

**Fig. 1:**
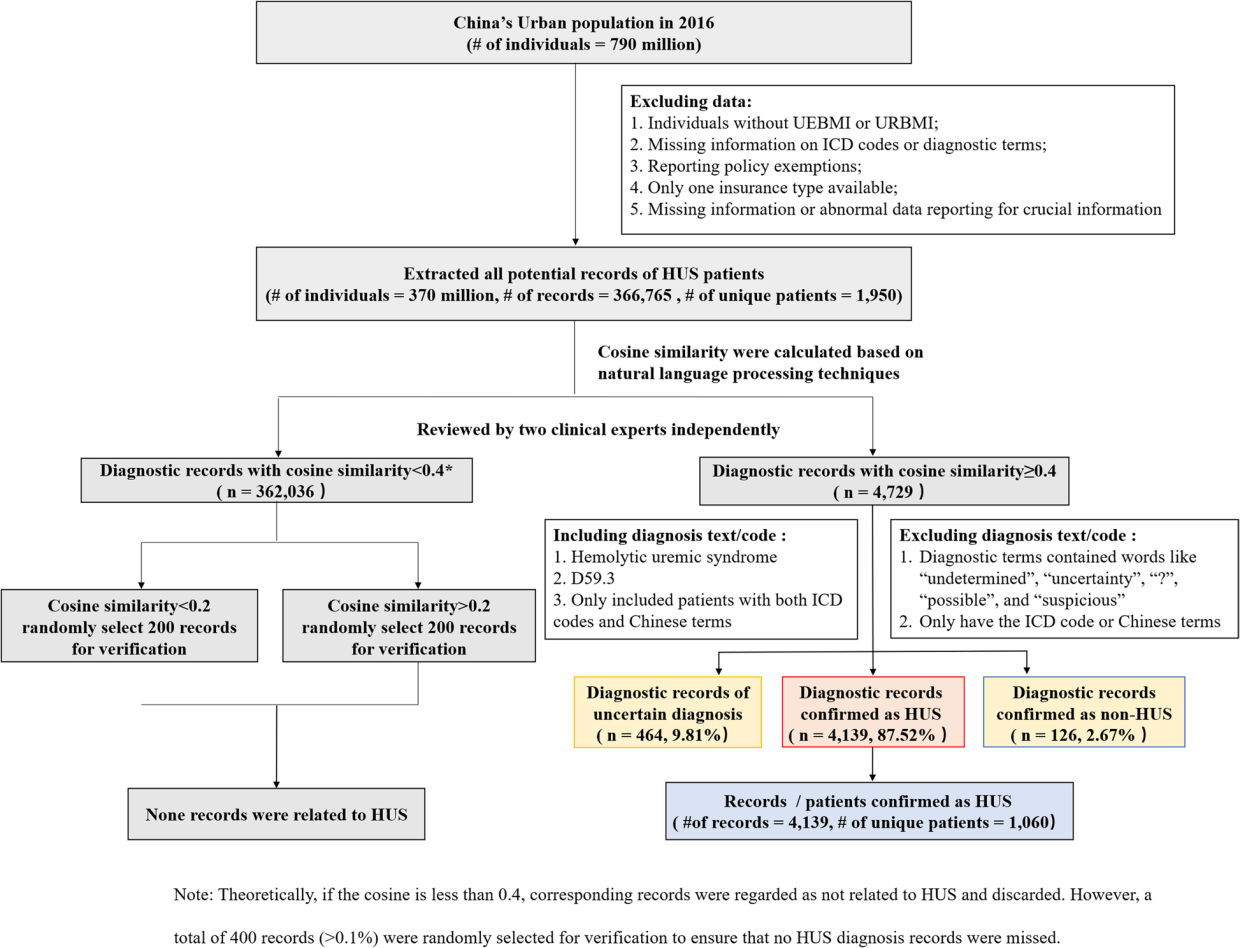
The flowchart of HUS case ascertainment

We identified potential HUS patients using ICD-9 codes (283.11, World Health Organization 10^th^, 1999), ICD-10 codes (D59.3, World Health Organization version 2010, 2010) and medical terms in Chinese. Natural language processing was utilized to standardize the text or codes. Diagnostic terms for each potential patient were independently reviewed by two clinical experts to confirm the diagnosis. If the diagnostic terms contained words like “undetermined”, “uncertainty”, “?”, “possible”, and “suspicious”, the patients were excluded; we only included patients with both ICD codes and Chinese terms. The flowchart is shown in Fig. 1. To further assess the impact of TMA on the results, we also extracted the potential TMA patients for the post hoc analysis using the diagnostic terms and ICD code (M31.1).

### Statistical analysis

All patients with STEC-HUS should undergo 2 to 5 years of follow-up to detect late-emerging sequelae, [12] and many patients with aHUS appear to have a life-long risk of recurrence [3]. We estimated the national incidence in only 2016 by setting up a four-year wash-out period (the longest period in our database) to reduce the impacts of prevalent HUS cases. The incidence estimate was also stratified by sex and age.

Incidences were calculated using a two-stage approach. First, we calculated the incidence in each province. The method was as follows: the denominator (*N*) used to calculate the annual incidence of HUS was the total number of patients in both the UEBMI and URBMI in each province during a given year. The numerator (*M*) was the estimated number of patients with HUS in the population used in the denominator in each province, with consideration of the issue of missing diagnostic values. In detail, the total enrolled population in each province was separated into three groups: subjects with no records of medical claims *(N*_*1*_), subjects with complete diagnostic information in the claims records (*N*_*2*_), and subjects with claim records but with missing diagnostic information (*N*_*3*_). The patients with incident HUS (*M*_*1*_) belonged to *N*_*2*_. However, N_3_ actually contained a certain number of patients with incident HUS (*M*_*2*_). Thus, we adopted a method based on Poisson regression to estimate *M*_*2*_. Then, a random-effects meta-analysis was used to pool the province-specific estimates to calculate the national average estimates. At this stage, the variance in the province-specific estimates was stabilized with the Freeman-Tukey double arcsine transformation [13].

The incidence was expressed per 100,000 person-years at risk. In addition, 95% confidence intervals (CIs) were also calculated assuming a Poisson distribution. Age-standardized rates were estimated using the Chinese 2010 census data as the standard population. Sensitivity analyses were conducted to assess the robustness of the results: (1) including only observed cases to assess the lower bounds of the rates and (2) excluding the top 10% of provinces with missing diagnostic information. We also calculated HUS-associated total costs per patient per year. Costs were discounted by consumer price index (CPI) in each year to 2016 and converted into US dollars based on the 2016 RMB to US dollar exchange rate (period average). The CPI and exchange rate were from the 2017 China statistical yearbook. Student’s t-test for continuous variables and the chi-square test for categorical variables were used for comparisons. All statistical analyses were conducted with Stata version 15.0, and 2-sided tests with *P* < 0.05 were considered statistically significant.

## Results

A total of 0.37 billion residents in 16 provinces were included in this study (Supplementary table 2). In addition, 1,060 patients received a confirmed diagnosis of HUS from 2012 to 2016, with a male to female ratio of 1.36:1. Most of the patients were Han Chinese (1001, 94.43%), with a disease onset in autumn and winter (606, 57.17%). In addition, the mean age (SD) of the total patients was 49.65 (15.82) and for male and female patients with HUS were 49.98 (15.66) and 49.21 (16.03) years, respectively (Table 1). Approximately 100 patients were diagnosed with TMA during the 5-year period.

**Table 1:** Selected characteristic of HUS patients grouped by sex

		Total	Male	Female	Comparison between sex	
					Statistics	*P* value
Number		1060	610	450		
Age, y					-0.782^a^	0.217
	Median age (Q_25_, Q_75_)	50 (39, 61)	50 (40, 61)	49 (39, 61)		
	Mean (SD)	49.65 (15.82)	49.98 (15.66)	49.21 (16.03)		
Age group, n (%)					9.926^b^	0.45
	< 1	5 (0.47)	2 (0.33)	3 (0.67)		
	1 ~ 5	5 (0.47)	4 (0.66)	1 (0.22)		
	6 ~ 11	7 (0.66)	4 (0.66)	3 (0.67)		
	12 ~ 17	3 (0.28)	2 (0.33)	1 (0.67)		
	18 ~ 29	94 (8.87)	49 (8.03)	45 (10.00)		
	30 ~ 39	162 (15.28)	89 (14.59)	73 (16.22)		
	40 ~ 49	250 (23.58)	143 (23.44)	107 (23.78)		
	50 ~ 59	242 (23.83)	149 (24.43)	93 (20.67)		
	60 ~ 69	178 (16.79)	99 (16.23)	79 (17.56)		
	70 ~ 79	94 (8.87)	61 (10.00)	33 (7.33)		
	> = 80	20 (1.89)	8 (1.31)	12 (2.67)		
Ethnicity, n (%)					6.040^b^	0.05
	Han	1001 (94.43)	585 (95.90)	416 (92.44)		
	Other	59 (5.57)	25 (4.10)	34 (7.56)		
Season, n (%)					2.189^b^	0.53
	Spring	189 (17.83)	102 (16.72)	87 (19.33)		
	Summer	265 (25.00)	159 (26.07)	106 (23.56)		
	Autumn	277 (26.13)	164 (26.89)	113 (25.11)		
	Winter	329 (31.04)	185 (30.33)	144 (32.00)		

^a^Student’s t-test

^b^Chi-square test

### HUS incidence

The national incidence of HUS was 0.66 cases per 100,000 person-years (95% CI:0.35–1.06) in 2016. Standardized to the Chinese 2010 census population, the total adjusted incidence rate for HUS was 0.57 cases per 100,000 person-years (95% CI: 0.19–1.18). The incidence rate for males was 0.68 cases per 100,000 person-years (95% CI: 0.36 to 1.11), which was slightly higher than that of females (0.58 cases per 100,000 person-years, 95% CI: 0.29 to 0.99). The highest incidence occurred in patients younger than 1 year old (5.08 cases per 100,000 person-years, 95% CI, 0.23–24.87) and the lowest incidence was approximately 0.10 cases per 100,000 person-years (95% CI: 0.03–0.20), which was observed in the 12 to 17-year-old group. Meanwhile, the incidences in patients younger than 5 years old, 15 years old and 18 years old were 0.38 cases per 100,000 person-years (95% CI: 0.13 to 0.75), 0.35 cases per 100,000 person-years (95% CI: 0.13 to 0.66) and 0.29 cases per 100,000 person-years (95% CI: 0.09 to 0.51), respectively. The incidence was the highest in autumn (0.87 cases per 100,000 person-years, 95% CI: 0.48 to 1.37), followed by winter (0.74 cases per 100,000 person-years, 95% CI: 0.33 to 1.32) (Table 2).

**Table 2:** Crude incidence of HUS grouped by sex, age-group and season (units: cases/100,000 person-years)

Groups		Incidence
Total		0.66 (0.35,1.06)
Sex	Male	0.68 (0.36,1.11)
	Female	0.58 (0.29,0.99)
Age group	< 1	5.08 (0.23,24.87)
	1 ~ 5	0.47 (0.15,0.97)
	6 ~ 11	0.23 (0.07,0.48)
	12 ~ 17	0.10 (0.03,0.20)
	18 ~ 29	0.60 (0.29,1.02)
	30 ~ 39	0.63 (0.29,1.09)
	40 ~ 49	0.47 (0.01,0.80)
	50 ~ 59	0.51 (0.21,0.93)
	60 ~ 69	0.65 (0.25,1.22)
	70 ~ 79	0.81 (0.30,1.56)
	> = 80	1.05 (0.42,1.95)
	< 5	0.38 (0.13,0.75)
	< 15	0.35 (0.13,0.66)
	< 18	0.29 (0.09,0.57)
Season	Spring	0.58 (0.26,1.02)
	Summer	0.58 (0.29,0.96)
	Autumn	0.87 (0.48,1.37)
	Winter	0.74 (0.33,1.32)

#### Costs of HUS

**Fig. 2:**
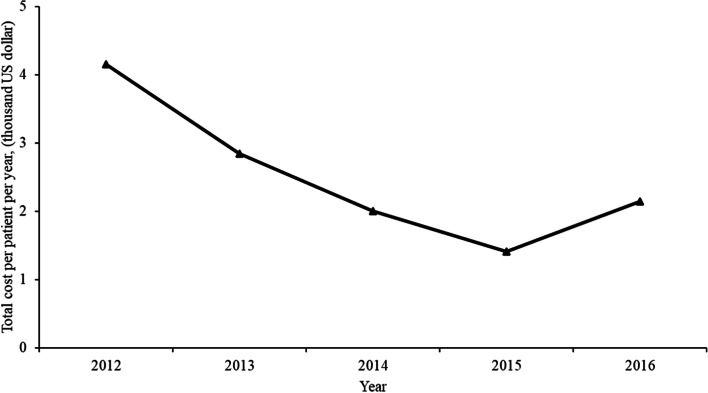
Medical expenses incurred by HUS patients in China from 2012 to 2016

The average total cost per patient over the study period was 2.15 thousand US dollars. The total cost per patient per year first decreased from 4.16 thousand US dollars in 2012 to 1.41 thousand US dollars in 2015 and then increased. By 2016, it was 2.15 thousand US dollars, which was close to the value in 2014 (shown in Fig. 2).

#### Sensitivity analysis

Considering only observed cases, the lower bound of the national incidence was 0.30 cases per 100,000 person-years (95% CI 0.19–0.43). The results calculated by excluding the top 10% of provinces (Shandong and Jiangxi) with missing diagnostic information was 0.50 cases per 100,000 person-years (95% CI 0.27–0.81), which was similar to the main results reported above.

## Discussion

In this large population-based study, we used a nationally representative database to calculate the incidence of HUS in China for the first time. The incidence rate of HUS was 0.66 cases per 100,000 person-years, with a peak incidence in children younger than 1 year old (5.08 cases per 100,000 person-years), and the incidence in males was slightly higher than that in females. The season with the highest incidence of HUS was autumn (0.9 cases per 100,000 person-years). It is also worth noting that the average total cost per patient was 2.15 thousand US dollars, and the large population base resulted in a large total cost.

A wide range of incidences (ranging from 0.07 to 10.5 cases per 100,000 person-years) of HUS have been reported in many countries, depending on the diagnostic criteria and populations studied [9, 14]. The incidence of HUS is positively correlated with the incidence of STEC infections, as STEC infection was reported to be the most common cause of HUS [8]. For instance, Argentina has one of the highest incidence rates of STEC and HUS in the world [14], while the incidence of HUS is much lower in Australia, where STEC outbreaks appear to less common than in many other countries [9]. The incidence rate in China is within the reported range and lower than that previously reported in Western countries [8]. The possible reasons for this observation are also related to the low incidence of STEC. First, our estimation of the incidence of HUS was extrapolated from the Urban Medical Insurance databases. There are some studies indicating that urban residents have a lower prevalence of STEC-HUS than rural residents [2, 15]. Cows, which are a reservoir of STEC, are much rarer in urban areas [16]. Second, with improvements in environmental hygiene, the incidence rate of STEC infection and STEC-HUS have decreased [17, 18]. Third, some authors have also reported that a higher socioeconomic status is associated with a higher risk of developing STEC-related disease and HUS, but the mechanism remains unclear [19, 20]. In addition, the genetic background plays a role in the susceptibility and severity of HUS. Some studies have shown differences in the genetic predisposition for the development HUS between black and white populations [2, 21]. However, the genetic predisposition for developing HUS in Asian populations is unknown, as there have been few large-scale epidemiological studies. Nevertheless, the mutations in completement factor H (CFH) and factor B (CFB) observed in aHUS patients are different between Asian and European populations, 0 %~ 17% vs. 20% ~ 30% and 3.4% ~ 18% vs. 1% ~ 4%, respectively [22, 23].

Consistent with previous studies, there was no significant sex difference [24, 25]. The 2016 incidence among children was 0.29 per 100,000, which is close to that in the United States in the same year (0.51 per 100,000) [18], while the peak age of onset in China is different from that in Western countries. In our study, the incidence rate was highest in children < 1 year of age (5.08 per 100,000 person-years). However, some previous reports from Europe and North America showed that the incidence rate was highest in children < 5 years of age (1.57 to 3 per 100,000 person-years, which is similar to the age-specific incidence pattern of STEC [24, 26]. We hypothesized that the higher incidence among Chinese infants may be attributable to genetic susceptibility, as hereditary aHUS must be considered in patients with a very young age of onset [22, 27–29]. In addition, some studies have shown that *Streptococcus pneumoniae* is the main cause of HUS in Chinese children (accounting for 60% ~ 100% of the cases), rather than STEC [30, 31]. This may be due to the lower pneumonia vaccination rates, and higher population density in China. However, these hypotheses require future investigation.

The peak incidence of HUS in our study occurred in autumn (0.9 cases per 100,000 person-years), followed by winter (0.7 cases per 100,000 person-years). However, the peak incidence in Western countries occurs in summer, which is also the peak period of STEC infections [2]. This significant seasonal difference also suggests that the proportion of STEC-HUS in urban China may be lower than that previously reported by studies conducted in Western countries, possibly owing to the relatively lower STEC infection rate in urban areas [15], genetic susceptibility, and regional background [21]. The difference in high-incidence seasons also needs to be further studied.

The total cost per patient per year was 2.15 thousand US dollars, which was higher than the national average annual medical care cost for urban residents (250 US dollars per patient). This may be mainly due to the high cost of some essential treatments for HUS. For example, the cost of a single plasma exchange or continuous renal replacement therapy (CRRT) session in China is approximately 1.50 thousand US dollars, and some HUS patients may need to receive multiple treatments to recover [1]. Furthermore, eculizumab, an anti-C5 antibody that is now considered a first-line treatment of HUS, is more expensive and may impose a significant economic burden on the China’s medical insurance system after its introduction in the near future. Therefore, it is critical to promote guideline-recommended therapy to improve prognosis and minimize the economic burden.

This study used a large nationally-representative sample of the Chinese mainland population, providing good estimates of the incidence and costs of HUS. However, the use of a medical insurance database still results in certain limitations. First, our data were extracted from urban populations. As STEC infections are more common in rural areas [2, 15], we may have underestimated the annual incidence of STEC-HUS in China. Second, HUS is a rare disease. The incidence rates of HUS in hospitals lacking diagnostic capabilities are probably underestimated. Some HUS patients may be diagnosed with TMA because of the inability to test for ADAMTS13. However, only approximately 100 patients were diagnosed with TMA during the 5-year period of this study. Compared with the 1060 patients who had a confirmed diagnosis of HUS, these 100 patients would not have had a substantial impact on the results. Last, the exact causes of HUS in China are still not known due to the limited availability of the relevant information. In particular, due to the unavailability of stool test results, we were unable to determine the exact proportion of patients with STEC-HUS infection in our study.

## Conclusion

In conclusion, this is the first population-based study of the incidence of HUS in urban China. Given the burden HUS imposes on patients and the medical insurance system, our investigation is of considerable importance for health care providers in China. In addition, our research also suggests that the aetiological structure of HUS in urban areas of China may be different from previous reports in some Western countries. To develop better preventive measures and treatments, such as vaccination against *Streptococcus pneumoniae*, appropriate antibiotic treatment and treatment with complement inhibitors, a national surveillance system and large-scale, detailed reports on HUS are needed.

## Supplementary Information

**Additional file 1:** **Supplementary table 1.** Sixteen provinces in China were included in this research.  **Supplementary table 2. **Basic characteristics of the populations in 16 provinces in China in 2016.

## Data Availability

Summarized health data on HUS can be accessed by contacting the National Insurance Claims for Epidemiological Research (NICER) Group, School of Public Health, Peking University. Contact email: 0016163159@bjmu.edu.cn.

## Abbreviations

HUS: Haemolytic Uraemic Syndrome
UEBMI: Urban Employee Basic Medical Insurance
URBMI: Urban Resident Basic Medical Insurance
ICD: International Classification of Diseases
CI: Confidence Interval
SD: Standard Deviation
CPI: Consumer Price Index
STEC: Shiga Toxin-producing *E. coli*
ESRD: End-Stage Renal Disease
CRRT: Continuous Renal Replacement Therapy
CFH: Complement Factor H
CFB: Complement Factor B
TMA: Thrombotic Microangiopathies
ADAMTS13: A Disintegrin and Metalloproteinase with a ThromboSpondin type 1 motif, member 13
DGKE: Diacylglycerol Kinase ɛ
TTP: Thrombotic Thrombocytopenic Purpura
