# Supplementary material for: Incidence and cost of haemolytic uraemic syndrome in urban China: a national population-based analysis
Source: BMC Nephrol. 2022 Mar 30;23:122. doi: 10.1186/s12882-022-02746-2 (PMC8969241; doi:10.1186/s12882-022-02746-2)
Supplement: Supplementary file 1 — Additional file 1: Supplementary table 1. Sixteen provinces in China were included in this research. Supplementary table 2. Basic characteristics of the populations in 16 provinces in China in 2016. [file 12882_2022_2746_MOESM1_ESM.docx]

## Supplementary table 1 16 provinces in China were included in this research.

| East | Anhui | Jiangsu | Jiangxi | Zhejiang | Shandong |
| --- | --- | --- | --- | --- | --- |
| North | Inner Mongolia | Shanxi |  |  |  |
| Northeast | Jilin | Heilongjiang |  |  |  |
| Northwest | Shaanxi |  |  |  |  |
| South-Central | Hubei | Henan | Hunan | Guangdong |  |
| Southwest | Yunnan | Chongqing |  |  |  |

**Supplementary table 2 Basic characteristics of the populations in 16 provinces in China in 2016**

|  | | **Total** | **UEBMI** | **URBMI** |
| --- | --- | --- | --- | --- |
|  |  |  |  |  |
| Total number (million) |  | 369.26 | 147.10 | 222.16 |
| Age, y |  |  |  |  |
|  | Mean (SD) | 37.57 (20.00) | 41.43 (15.34) | 35.02 (22.20) |
| Age group, n (%) |  |  |  |  |
|  | <1 | 0.64 (0.17) | 0.01 (0.01) | 0.64 (0.28) |
|  | 1~5 | 13.96 (3.78) | 0.13 (0.09) | 13.82 (6.23) |
|  | 6~11 | 22.38 (6.06) | 0.11 (0.08) | 22.27 (10.02) |
|  | 12~17 | 22.68 (6.14) | 0.30 (0.19) | 22.38 (10.08) |
|  | 18~29 | 87.87 (23.80) | 40.04 (27.22) | 47.83 (21.53) |
|  | 30~39 | 57.41 (15.55) | 35.13 (23.88) | 22.28 (10.03) |
|  | 40~49 | 60.93 (16.50) | 30.89 (21.00) | 30.04 (13.52) |
|  | 50~59 | 45.30 (12.27) | 19.92 (13.54) | 25.38 (11.43) |
|  | 60~69 | 32.88 (8.90) | 12.08 (8.21) | 20.80 (9.36) |
|  | 70~79 | 16.33 (4.42) | 5.82 (3.96) | 10.51 (4.73) |
|  | >=80 | 8.88 (2.41) | 2.67 (1.82) | 6.21 (2.79) |
| Sex, n (%) |  |  |  |  |
|  | Male | 192.57 (52.15) | 82.37 (55.99) | 110.20 (49.60) |
|  | Female | 176.69 (47.85) | 64.73 (44.01) | 111.96 (50.40) |
| Ethnicity, n (%) |  |  |  |  |
|  | Han | 310.42 (84.07) | 133.49 (90.75) | 176.93 (79.64) |
|  | Others | 58.84 (15.93) | 13.61 (9.25) | 45.23 (20.36) |

Abbreviations: UEBMI, urban employee basic medical insurance; URBMI, urban resident basic medical insurance.
